# Editorial

**Published:** 1867-12

**Authors:** 


### Editorial.

---

### LEGAL.

WE hope our professional brethren in the State will bear in mind the important work they now have in hand, viz.: the securing legal recognition and protection, and appreciate the importance of immediate action. Let the petitions in their hands be immediately filled with signatures, and information given to all whom it may concern.

There is no doubt that by prompt and concurrent effort on the part of the Dentists in the State, the desired law could be obtained in one month. Then let all take hold at once and accomplish it, and not have the matter hanging fire as it has hitherto done.

Kentucky will certainly succeed this winter in obtaining such a law, and very probably several other neighboring States, and shall Ohio who took the initiative in this matter lag behind, and suffer the ill consequences of other States preceding in this work? Professional brothers, your reply to this, I trust, will be by deeds. T.

### MEETINGS OF SOCIETIES.

THE regular meeting of the Mad River Dental Association was held in Xenia, on Monday, December 24th, at the office of Dr. Geo. L. Paine.

The meeting was well attended, and fully enjoyed by those present; various subjects were discussed, and in such a manner as to show that improvement and progress is being constantly made. The discussions upon the question, "What conditions and circumstances modify the results of operations upon the teeth?" was very interesting indeed, and called forth a train of thought that by many was not before clearly apprehended.

This Society is one of the most active in the State, always meets promptly and has a good attendance. It was one of the first to act promptly and squarely up to the enforcement of the Code of Ethics, and by that act added much to its prestige and organic strength.

The next meeting will be held in Hamilton in April, when we confidently expect to see the largest meeting it has ever held.

T.

---

The annual meeting of the Ohio State Dental Association was held in Columbus, Dec. 24th, during the day and evening.

The attendance was not large,—the time was not favorable, owing to its proximity to the holidays,—yet the meeting was a spirited one; much business was transacted, and several topics pertaining to practice were discussed.

It was resolved to industriously labor for the procurement of a law regulating Dental practice in the State. The necessity for such a law is becoming more and more apparent every year.

By the action of the Association, the course pursued by the Executive Committee, in the defense of the profession against the unjust claims of the Goodyear Dental Vulcanite Company, was most heartily approved, and the committee were requested to press forward in the defense, as in their wisdom shall seem best.

By resolution the semi-annual meeting was suspended, and it is understood that hereafter the society will hold only annual meetings, when it is supposed the attendance will be much larger, and perhaps in the aggregate quite as much business accomplished as by two sessions annually. T.

### INTERESTING.

We give place to the following items, feeling that they contain matter of gratification to the profession.

The statements of Drs. Berry and Smith are worthy of attention, they having made thorough test of the new material.

In the U. S. Court on Monday last, in the case referred to below, the complainants finding themselves nonplussed, asked for further time for preparation, and the hearing for infringement was fixed for the 20th of February next, at which time the Executive Committee hope to be able to fully sustain the interests of the profession; and the Committee trust that the profession will sustain them in this contest, and promptly too, and not, now that things look bright, withhold that aid and assistance which is absolutely necessary to success. T.

---

LEGAL CONTEST WITH THE DENTISTS.—A motion was made in the United States District Court in the cases of Goodyear *et al.* vs. Taft, Berry, Smith, and seven others, for a preliminary injunction, based upon the late decision of Judge Nelson in the Vulcanite case. The defendants replied that they had abandoned the Goodyear compound, and were now using a rubber prepared by the Porter Manufacturing Company, under the patent granted to Edward L. Simpson, October 16, 1866. Affidavits were filed to prove that this compound contained only two and a half ounces of sulphur to a pound of rubber; the minimum fixed by the Goodyear patent being four ounces. Upon the day appointed for the hearing, both parties appearing, the complainant's counsel withdrew his motion, and abandoned the application for an injunction in all the cases.

---

A WORD RESPECTING RUBBER.—Having used all the different preparations of Dental rubber in the market, I take pleasure in saying, that after about six weeks exclusive use of the "Improved Dental Rubber," manufactured by A. R. Hale, under Simpson's patent, issued October 16, 1866, I am highly gratified, and prefer it to any I have seen.

The Simpson rubber requires more heat or longer time than the Goodyear for vulcanizing. If the "Directions for Steaming" accompanying the Simpson gum are followed, it is of a lighter shade of color than if steamed a shorter time; but as I regard this of little importance, I run up the mercury to 330 or 335, and steam from fifty to fifty-five minutes. Of course the necessary time may differ in different vulcanizers according as they differ in preventing escape of steam.

167 Race street, Cincinnati, A. BERRY.
January 6, 1868.

---

EDITORS REGISTER:—Feeling it a duty for all members of the profession to call attention to improvements in Dental work, I would say that I have been using the rubber made by A. R. Hale, under the Simpson patent, and find it equal to the best and superior to most of the rubber used for Dental purposes; and would advise all Dentists to give it a trial, and think they will be highly pleased with it. H. R. SMITH.

### OBITUARY.

At a regular meeting of the Albany and Rensselaer Co. Dental Association, held at Troy, N. Y., December 10th, 1867, the following preamble and resolutions were adopted:

Whereas, It has pleased the Almighty Ruler, in his wise providence, to remove by death from our midst Dr. D. F. Benne and Dr. Robert Nelson, both of Albany; therefore be it

*Resolved*, That in the death of Drs. Benne and Nelson, this Society has lost two valued members, much beloved and highly appreciated by us for their great skill and zeal in the interest of the profession.

*Resolved*, That the citizens of Albany and vicinity have sustained a severe loss in the death of these gentlemen.

*Resolved*, That to their immediate friends this Society tenders its respectful and cordial sympathy, and that these resolutions be published in the Albany and Troy daily papers and the Dental Journals, and a copy, signed by the President and Secretary, be presented to the families of the deceased.

Drs. H. H. Young, J. C. Austin, W. F. Winne,
*Committee on Resolutions.*

W. F. Winne, *Secretary*. S. D. French, *President*.

---

At a meeting of the Students of the Ohio College of Dental Surgery, to take action in reference to the death of one of their best members, Alfred A. Sears, M. D., of Mount Carmel, Illinois, the following resolutions were unanimously adopted:

Whereas, An allwise Providence having seen fit to remove from among us, after a short and painful sickness, our fellow student, A. A. Sears, M. D., therefore,—

*Resolved*, That in the death of Dr. Sears, the Dental profession has lost an able and conscientious member, and the college an indefatigable student.

*Resolved*, That in his death society has lost one of her noblest hearts, and his friends a soul that was devotion and truth.

*Resolved*, That we the Students of the Ohio College of Dental Surgery, deeply sympathize with his bereaved parents in this the sad hour of their affliction.

*Resolved*, That a copy of the above resolutions be sent to the parents of Dr. Sears, to the Jefferson Medical College of Philadelphia, and to the Editors of the Register for publication.

R. J. O. Mahoney, *Secretary*. R. H. Lawrence, *Chairman*.

